# Working Relationally with Adolescents Who Have Experienced Complex Interpersonal Trauma: an Interpretative Phenomenological Analysis

**DOI:** 10.1007/s40653-021-00349-x

**Published:** 2021-03-24

**Authors:** Nicola Amari, Adam Mahoney

**Affiliations:** 1 Department of Psychology, School of Health & Life Sciences, Glasgow Caledonian University, Glasgow, UK; 2 School of Applied Sciences, Edinburgh Napier University, Edinburgh, UK

**Keywords:** Counselling psychology, Complex trauma, Adolescence, Use of self, Person of the therapist

## Abstract

The study explores practitioners’ experiences of working with adolescents with complex interpersonal trauma. Five mental health professionals were recruited via purposive and snowball sampling. Semi-structured interviews were conducted, audio-recorded and transcribed. Interpretative phenomenological analysis was employed to identify themes related to the research question. Three superordinate themes emerged: “The distance-relationship dynamic”; “The unknown self”; “Practitioners’ presence”. Participants reported the fragility of their relationships with clients, enhanced awareness of their professional and personal identity, and highlighted the importance of meeting their clients as persons. Results are discussed within the literature that considers the role of the person of the therapist to foster therapeutic change. Future research could explore the role of therapeutic distance, the interplay between transference-countertransference and attachment dynamics, and the relevance of practitioners’ playfulness with traumatised youth. Finally, this study proposes a model of use of self as “compassion-in-action” to express practitioners’ ‘response-ability’ to their clients’ trauma as commitment to social justice.

Research on the person of the therapist suggests that the ability to provide empathy (Elliott et al. 2018), positive regard (Farber et al. 2018), and genuineness (Kolden et al. 2018) fosters clients’ process of positive change (Norcross and Lambert 2018; Rogers 1957). Specifically, combining theoretical and empirical findings, the person of the therapist has been conceptualised as the therapeutic use of self, including self-disclosure and transparency in addition to the interplay of transferential/countertransferiential dynamics with clients (Hill et al. 2018; Knight 2012; Kolden et al. 2018). Despite the challenges to demonstrate a causal relationship between the qualities that are interwoven in the practitioner’s self and therapy outcomes (Norcross and Lambert 2018), or to account for the differences in clients’ presentations and culture (Hall et al. 2016), the dynamic of practitioners’ and clients’ characteristics arguably affects the effectiveness of psychological treatments. Consonantly, the encounter between practitioners and clients whereby they experience caring for each other on a person-to-person level has been positively correlated to clients’ recovery (Gelso et al. 2018). Thus, a focus on the person of the practitioner may help pinpoint those features that contribute to the enhancement of the therapeutic alliance with clients (Delgadillo et al. 2020; Heinonen and Nissen-Lie 2020).

The significance of the person of the therapist appears to be more relevant the more complex the client’s presentation (Castonguay and Hill 2017). Arguably, this would be the case of clients with multitude traumatic experiences who can evoke strong feelings related to countertransference (Wolstein 1988). Accordingly, clients with severe mental illnesses have been found to have a history of interpersonal trauma resulting from abuse and neglect within relationships during their formative years (Mauritz et al. 2013). Specifically, the pervasive impact of developmental trauma on clients’ self-concept, attachment, behavioural and emotional regulation has elicited the refinement of trauma-related disorders, with the addition of complex post-traumatic stress disorder (CPTSD) to the eleventh edition of the International Classification of Diseases (ICD-11; Cook et al. 2005; Rosenfield et al. 2018). As opposed to single-event trauma, CPTSD refers to a cluster of enduring reactions (e.g. emotional dysregulation; sense of self eroded by feelings of shame, worthlessness and failure; severe disturbances in relationships) which can be caused by the exposure to multiple traumatic events of an interpersonal nature (Maercker et al. 2013). However, whilst a focus on symptomatology risks objectifying clients’ experiences, therapist-focused research can highlight practitioners’ contribution to facilitating clients’ sense of connectedness, thus providing an experience of interpersonal healing (Badenoch 2018; Barkham et al. 2017; Frewen and Lanius 2015; Siegel and Solomon 2013). Consequently, an understanding of practitioners’ beliefs, values, feelings, and reactions may be relevant to the development of effective therapeutic interventions in trauma work (Murphy and Joseph 2013; Norcross and Wampold 2019).

The awareness of the developmental disruptions that may occur as a consequence of interpersonal chronic trauma challenges clinicians to deliver a flexible approach that is empirically supported, centred on the therapeutic relationship, and promotes resilience (Lawson and Quinn 2013; Steenbakkers et al. 2018; Treisman 2017). Consonantly, intersubjectivity theory provides a framework to appreciate the relational dynamics occurring within clinical practice (Rasmussen 2005), such as vicarious traumatisation (Bercier 2013; Ireland and Huxley 2018), compassion fatigue (Sodeke-Gregson et al. 2013; Zerach, 2013), transference/countertransference (Gartner 2017), and vicarious resilience (Hernández et al. 2007). Furthermore, qualitative studies can enrich the understanding of practitioners’ experiences whilst working with traumatised populations (Mattar and Vogel 2014). Specifically, a focus on child and adolescent research has been advocated by international bodies (International Society for Traumatic Stress Studies [ISSTS], 2017) in line with the development of early interventions to counteract the effects of adverse childhood experiences (Felitti et al. 1998) and intergenerational trauma (Isobel et al. 2019). Resonating with the acknowledgement that the attention to young people’ perspectives can optimise treatment guidelines (van Wesel et al. 2014), practitioners’ accounts can illuminate the process of the therapeutic relationship from the therapist’s perspective as there might be specific systemic, emotional and experiential dynamics (Lowe 2016). For instance, despite the vulnerability to secondary trauma, practitioners may experience a sense of fulfilment and experiment with an attitude of playfulness whilst relating to young people (Wheeler and McElvaney 2018). Conversely, a lack of an interagency support system in their organisational context would undermine clinicians’ confidence in providing a safe therapeutic environment (Edwards and Karnilowicz 2013; McElvaney and Tatlow-Golden 2016). Alternatively, as a subtype of countertransference, vicarious parenting would capture practitioners’ parental feelings towards their young clients whilst dealing with systemic difficulties (Fixsen et al. 2019). Therefore, by enhancing the understanding of the therapist’s use of self with specific clients’ presentations, therapist-focused qualitative evidence may optimise treatment guidelines (Heinonen and Nissen-Lie 2020).

Embracing an intersubjective perspective, counselling psychologists have advocated the importance of working relationally with complex trauma, thus acknowledging the person’s environment and relationships (Walsh et al. 2013). As research shows differences even when clinicians adhere to standardised treatments (Norcross and Lambert 2018), the appreciation of the person of the therapist begs the question as to how practitioners use their ‘self’ in their therapeutic work with clients. In the UK, the Health and Care Professions Council ([HCPC], 2015) sets as a distinctive standard for counselling psychologists the critical reflection on the use of their ‘self’ in the therapeutic process, thus emphasising the importance of personal therapy, supervision, and continuing personal development. Consonantly, drawing from the results of their interviews with practitioner psychologists, Sleater and Scheiner (2020) have proposed a model of the use of self within a counselling psychology framework. Sleater and Scheiner’s model of the use of self comprises the symbiosis between the therapists’ awareness and wellness, which would enrich the connection between the therapist’s and the client’s way of being in a mutual relationship. However, research on the person of the therapist appears to not have been anchored to specific clients’ presentations (Castonguay and Hill 2017). Accordingly, in addition to the call for developmentally sensitive trauma-focused interventions (Ford 2015; Kliethermes et al. 2014) and assessments (Denton et al. 2017), child and adolescent research should explore how practitioners’ use of self may differ depending on clients’ developmental stages. As counselling psychology appreciates the primacy of the therapeutic relationship (Woolfe 2010) with a commitment to social justice (Cutts 2013), its relational approach to trauma work (Jordan 2010) appeals to practitioners to reflect on how they use their ‘self’ to relate to the ‘self’ of clients who have been exposed to complex trauma within the context of an interpersonal relationship (Fisher 2017; Norcross and Wampold 2019). Consequently, child and adolescent qualitative research can reveal nuances in the practitioners’ use of self in their therapeutic trauma work.

Within qualitative methodologies, Interpretative Phenomenological Analysis has been applied to explore practitioners’ lived experience of their therapeutic work and participants’ sense of self (Shinebourne 2011; Sleater and Scheiner 2020; Smith, 2004). For instance, whilst advocating the integration of a relational approach into technical interventions (Schottenbauer et al. 2008), Malcolm and Golsworthy (2020) explored counselling psychologists’ experiences of working relationally with clients who experienced abuse, highlighting practitioners’ interpersonal skills to facilitate therapeutic engagement and successful treatment outcomes. Similarly, by considering practitioners who work therapeutically in the field of complex interpersonal trauma, this study builds on existing qualitative trauma literature (van Wesel et al. 2014). Whilst resonating with a multidisciplinary approach to mental health, this study also responds to the call for child and adolescent trauma research and focuses on an understudied population, namely adolescence (Saxe et al. 2007). Specifically, this study aims to refine the understanding of the person of the therapist by investigating practitioners’ use of self in their work with repeated interpersonal trauma in adolescence (Briere and Lanktree 2011; Ford and Courtois 2013). Consequently, this study will investigate the lived experiences of practitioners working with adolescents with a symptomatology associated with CPTSD (i.e. repeated interpersonal trauma). Accordingly, the objectives of this study are:

- To explore practitioners’ experience of the relational dynamics with adolescents who present with complex interpersonal trauma;
- To explore practitioners’ experiences of building a therapeutic relationship with traumatised adolescents;
- To clarify the role of the person of the practitioner by exploring practitioners’ understanding of how they use their ‘self’ in their trauma work with adolescents.

The study will be exploratory to elicit insights on how practitioners support this population. Therefore, the main research question is:

- What are practitioners’ lived experiences of working with CPTSD in adolescents?

## Methods

### Design

IPA (Smith 2011) was employed to explore practitioners’ experience of the therapeutic process as shown in previous research informed by a phenomenological paradigm (Malcolm and Golsworthy 2020; Sleater and Scheiner 2020). Furthermore, IPA’s double hermeneutic (Smith 2011), which encompasses both the participant’s sense making of their experience and the researcher’s sense making of the participant’s experience, resonates with intersubjectivity theory, which acknowledges the subjectivities involved in the research process, namely the participants and the researcher (Malcolm and Golsworthy 2020). Accordingly, rather than asserting objective truths, the epistemology of IPA is underpinned by a phenomenological-existential paradigm, thus asserting that meaning is co-constructed. Consequently, the choice of IPA methodology illustrates a coherence between research question and the theoretical perspective adopted (Yardley 2000).

### Participants

The sample comprised five practitioners defined as mental health professionals with a theoretical understanding of psychotherapeutic approaches to inform their practice. The sample size was set to allow an in-depth analysis according to the methodology and the study timescale (Smith et al. 2009). Moreover, given the multidisciplinary support available to young people in children and adolescents mental health services (Dogra et al. 2017), this study included practitioners from different professional backgrounds (Walker 2003). Echoing the pluralistic value base of counselling psychology practice (Challoner and Papayianni 2018), participants’ breadth of experiences ranged from educational, forensic, counselling psychology to social work, psychoanalysis, and dyadic developmental psychotherapy, thus ensuring sensitivity to different perspectives (Yardley 2000; see Table 1 for participants details).

**Table 1:** Participants details

Participant Pseudonym	Profession	Background & orientation
Gordon	forensic psychology	cognitive-behavioural therapy schema therapy compassion-focused therapy residential work
Helen	counselling psychology	person-centred therapy cognitive-behavioural therapy gestalt therapy play therapy
Judith	dyadic developmental therapist	person-centred therapy educational psychology family therapy residential work trainer
Mark	service manager (children & adolescents trauma service)	social work Eye movement desensitization and reprocessing therapy play therapy polyvagal theory trainer
Sarah	child and adolescent analyst	object relations theory - kleinian psychoanalysis

Despite this professional diversity, sample homogeneity required practitioners to: be fluent in English; be fit to practice; have a minimum of two years of experience post-qualification (Schwebke 2014) work with adolescents (aged 12–17; Minnis and Del Priori 2001) presenting a symptomatology associated with complex interpersonal trauma (CPTSD), as defined in the ICD-11 (Cloitre et al. 2018), thus ensuring sensitivity to context and rigour (Yardley 2000). Purposive and snowballing sampling was adopted (Smith et al. 2009) to approach practitioners and flyers with the research advert and the researcher’s contact details were sent to child and adolescence psychological trauma services.

### Procedure

Ethics approval was obtained through the university ethics committee. An interview schedule (see Appendix) was developed drawing from previous research looking at practitioners’ experiences of the therapeutic process with survivors of abuse and their work with traumatised adolescents (Malcolm and Golsworthy 2020; Schwebke 2014; Sleater and Scheiner 2020). The interview was semi-structured to ensure that participants could address similar topics, whilst preserving their idiographic perspective (Smith et al. 2009). Practitioners were asked to reflect on the use of self in the therapeutic process and on what they meant by using their ‘self’. The common first interview question was: Can you begin by telling me about your experience of working with complex interpersonal trauma in adolescents? To demonstrate commitment (Yardley, 2000), a pilot interview was completed to refine the appropriateness of the questions. Participants were given an information sheet and were made aware of their voluntary contribution to respect their autonomy (British Psychological Society, [BPS], 2014). Upon obtaining consent, participants were interviewed over online platforms for approximately sixty minutes (Smith et al. 2009). A semi-structured interview was conducted though clarification was sought to check the researcher’s understanding matched participants’ idiosyncratic perspective. Specifically, by clarifying the meaning of responses with their implications and asking follow-up questions, an iterative process was followed to ascertain participants’ agreement with the researcher’s account before proceeding to following questions. Furthermore, participants were made aware that they could withdraw at any point/refuse to answer questions. Interviews were recorded with a dictaphone. A master copy for safe storage and a working copy of the audio files was made using hard drive and email storage to securely dispose of data, which was password protected and accessible only by the researcher to preserve confidentiality (BPS 2014). Identifiable information was removed as interviews were transcribed verbatim and participants were assigned a pseudonym for anonymity (BPS 2014). Following the interview, participants were provided with a debrief sheet including support resources and the contact details of the researcher and the supervisor to reduce risk of harm and ensure accountability (BPS 2014; Yardley 2000).

### Data Analysis

The analysis followed the steps outlined by Smith (2011). Each transcript was read several times to aid familiarity with the content. Relevant passages were underlined and then descriptive, linguistic and conceptual comments were added, which were followed by additional questions to interrogate the content (Smith 2011). Subsequently, provisional codes were identified and assigned to emergent themes. The same process was followed for each transcript. Recurrent themes were selected as reported by at least three participants and remaining codes were disregarded. Further reviewing of the codes was undertaken to ensure relevance with participants’ general and idiosyncratic accounts. To enhance rigour and transparency (Yardley 2000), the researcher’s supervisor read and analysed one full transcript, adding further comments and checking the researcher’s comments, providing subject-matter expertise due to clinical and research experience in the field of CPTSD. Saturation of data was achieved by comparing the themes emerged from the interviews and confirmed by reordering the sequences of the interview to consider any variance (Constantinou et al. 2017). Additionally, credibility checks were offered by the researcher’s supervisor for the appropriateness between the quotes and the themes as a quality indicator (Elliott et al. 1999). Superordinate and subordinate themes were chosen as reflecting the research focus, thus adhering to the coherence principle in qualitative research (Eliott et al. 1999). Finally, given the sample size, each theme was supported with extracts from three participants to allow in-depth analysis (Smith 2011).

### Reflexivity

Research question was driven by the researcher’s exploration of the use of self as part of training in counselling psychology (HCPC 2015). Whilst the humanistic value base could have impacted the interpretative activity, IPA appreciates the researcher’s analysis as part of the hermeneutical process. A research diary was kept to record initial biases and assumptions that could interfere with the interpretation to facilitate a process of bracketing. Further, the researcher’s supervisor, with expertise in forensic psychology and complex interpersonal trauma, could offer a different perspective by conducting an independent analysis with commentary.

## Results

Three superordinate themes were identified (see Table 2). The superordinate themes will be discussed to capture the significance of the person of the practitioner and the use of self in trauma work with adolescents.

**Table 2:** Superordinate themes (3) and subordinate themes (7)

Superordinate theme	Subordinate theme
1. The distance-relationship dynamic	a. Connectedness b. Distance
2. The unknown self	a. Counter-transference b. Supervision c. Identity
3. Practitioners’ presence	a. Authenticity b. Playfulness

### Superordinate Theme 1: The Distance-Relationship Dynamic

The first superordinate theme explored the relational dynamic experienced by practitioners towards their adolescent clients, illustrating an oscillation between connectedness and a sense of distance. Whereas feelings of connection appeared to expand practitioners’ sense of self, distance evoked confusion about a looming therapeutic rupture. Thus, practitioners’ ability to hold this dialectical tension offered an inner compass in their therapeutic work.

Participants’ accounts of the therapeutic relationship with adolescents who had experienced complex trauma comprised a variety of responses, from positive feelings to anxiety, sadness and uncertainty. Being in the therapeutic relationship transcended temporal and spatial limitations as participants reached a sense of emotional attunement with clients. “Helen” attempts to verbalise this experience.

Joy, happiness, […] a sense of belonging […], trust […], not […] trying to find the way in but rather being calm and staying beside. A sense of distance […] in a sense of respect rather than distance in a sense of being far away […] there can still be […] space for you and space for me. (Helen)

Helen’s feelings of happiness and connection appear to relate to being allowed to be her ‘self’ with no pretences as if the therapeutic relationship ensued as a side effect of the practitioner’s surrender to the other’s presence, whilst expressing the practitioner’s genuineness. There seems to be a sense of acceptance in not trying to connect with clients because Helen is beside them wherever they are, thus hinting at being in a relationship as an ontological principle. Therefore, awareness of the other becomes acceptance as the therapeutic relationship is a shared space wherein two subjectivities meet without fearing that their space will be taken over by the other.

However, without the mutual acknowledgement of being in a relationship, the sense of distance from clients might be disconcerting:

I have a sense of either being quite close to tears or […] just floating around in space. You know, I have no connection with [the client] […]. This terrible sense of lostness and nobody being on an […] emotionally attuned level […]. They're somewhere else... Or they're so far away […], it's like throwing lifelines... to them... But they haven't actually caught anything. (Sarah)

“Sarah” describes the experience of disconnection with her adolescent clients. With no sense of direction, Sarah is surrounded by the unknown with nothing that anchors her, arguably hinting at a lack of belonging. As Sarah appears to struggle in relating to the young person, there is an unsettling feeling of not knowing where she is, which provokes an existential angst that arguably threatens her identity. Although the experience of trauma has arguably made the young person inaccessible to ensure safety, Sarah seems to be challenging the existential threat of meaninglessness by holding to a sense of power coming from the belief that the therapeutic relationship can be lifesaving. Seemingly transferring to clients her need to escape this unbearable suffering, Sarah seems to be questioning an insoluble existential separateness. However, by entering the therapeutic relationship, the other would offer meaning to Sarah’s endeavours to connect. Thus, with no relationship, Sarah’s self might not survive and sink in the abyss of the unknowing.

The distance-relation dynamic reflected participants’ acknowledgement of the uncertainty of their trauma work. The vulnerability of the therapeutic relationship to ruptures appeared to be related to the complexity of young people’s trauma as participants emphasised the importance of owning mistakes to instil trust. For instance, practitioners mentioned how developmental transitions or life changes might interact with their clients’ emotional and behavioural presentations which would reverberate within the therapeutic relationship. Accordingly, practitioners identified the reparation of the relationship as a constant feature of their clinical work. By admitting that ‘*errare humanum est’* in a relationship that is vulnerable to risk, practitioners’ ethical awareness emerged through compassion as acknowledgement of their shared humanness with clients. Furthermore, practitioners mentioned the organisational context of their practice and the context inhabited by the young person, highlighting how this wider network would impact their clinical work.

Overall, participants used ‘sight-related’ words whilst referring to clients, almost implying the presence of a barrier between them and the adolescents, thus suggesting the difficulty in engaging with this traumatised population. “Judith” elaborates on what seeing someone means:

I aim to […] give them my experience of seeing them […] - [to see them] is to love them […] no matter what […], to see the individuality, the specialness, the uniqueness […] and also to be there […] through difficult things. (Judith)

Judith’s intention is to offer young people her felt understanding of their otherness, which resonates with emotional attunement. Judith’s acknowledgement of the clients’ alterity beyond expectations becomes the confirmation that they are worthy of unconditional love and of having someone who is there for them through their traumatic experiences.

Consequently, participants’ experience of a ‘distance-relationship’ dynamic with their clients highlighted the preciousness and the fragility of their connection, whilst confirming the client’s radical alterity.

### Superordinate Theme 2: The Unknown Self

The second superordinate theme emerging from the participant’s account was the unveiling of a part of their self at the edge of their awareness. Participants engaged in a reflexive process that enabled them to become conscious of the personal impact, understanding, and change experienced whilst working with traumatised adolescents. Thus, practitioners emphasised the dangerousness associated with not knowing their ‘self’ as detrimental for their clients, thus linking self-awareness to the ethical principle of non-maleficence.

Firstly, participants framed their emotional, cognitive, and bodily reactions within a transference-countertransference dynamic, suggesting that their clients’ presence enhanced their self-awareness. Specifically, arguably given the developmental stage of their clients, practitioners shared a reflection on their own attachment history, whilst hinting at a parental-protective role. Voicing their reflection-in-action, practitioners illustrated a bidirectional process directed inwardly to their ‘self’ and outwardly towards the client’s otherness. Instead of a self-referential processing, practitioners’ self-awareness guided them to acknowledge their clients, thus acquiring an ethical connotation. “Mark” expands on the meaning of self-awareness in the practitioner’s therapeutic role:

my job is to bear witness to other people's suffering. […] And that's potentially dangerous for the practitioner. So that's why it's really important that we recognise what's our stuff and what's someone else's stuff and not […] to develop secondary post-traumatic stress […]. […] without the compassion, without the idea that you can contribute to the reduction of suffering [...] I don't see how this […] work would be possible […]. (Mark)

Mark states that his role is to acknowledge the existence of the young people’s suffering. Identification with the otherness and meaninglessness of this suffering would arguably be the danger for the practitioner. Seemingly referring to a transference-co(unter)transference dynamic, Mark cautions about vicarious traumatisation. Accordingly, self-awareness would counteract the risk of enmeshment with the young person’s difficulties whilst showing the practitioner’s personal involvement. Faced by the threat of meaninglessness of human suffering, Mark’s self-awareness becomes his ‘response-ability’ to the client’s suffering and equates to compassion as endeavour to reduce such suffering. Thus, Mark’s words arguably evoke his need for belongingness and a transpersonal ‘self’ that becomes aware of its inherent relational nature.

Secondly, practitioners’ response-ability suggests that, rather than a solitary expedition, self-awareness is a relational process that attests the ethical commitment to provide clients with a safe therapeutic relationship. Consonantly, participants expressed how the process of knowing their ‘self’ underpins the principle of beneficence and is ongoing because open to change in the encounter with otherness. Accordingly, participants resort to supervision to enhance their self-understanding and unveil previously unknown dimensions of self, as captured by Helen.

I really struggled […] and I had to use supervision […] to find out what that struggle was […] and my supervisor challenged me on that […] the struggle was that there was a blind side […]. I didn't see or I couldn't see but I didn't see...em.. and that was […] my thinking and my behaviour and […] my reaction […] And that needed to be challenged. (Helen)

Helen emphasises the efforts involved in the process of reflecting on one’s self. Supervision becomes a necessity because the relationship with her client signalled something unacknowledged in Helen. The “blind side” may allude to one aspect of Helen’s self of which she perceives the presence but that she cannot yet identify. Helen sounds uncertain as if there were an obstacle that prevented her from seeing this unknown part of her ‘self’. From its concealed position, this blind side seems to resonate with an understanding of the unconscious as an autonomous force that influences Helen’s thoughts, behaviours, and reactions. Thus, supervision becomes an ally in Helen’s seemingly inner conflict where the “blind side” would eventually be conquered and become known. Helen’s dichotomous representation of the self appears to comprise a conscious and an unconscious dimension. Therefore, knowing one’s self becomes the process of conquering the uncharted territory over which the self can expand. Perhaps, the unconscious self – as part of the ‘self’ of which the practitioner was not aware – had presented itself as a struggle in its willingness to be acknowledged, provoking the practitioner’s response and mirroring the relationship dynamic between the practitioner and the client.

Thirdly, with its relational and ethical foundation, participant’s self-awareness transpired from their reflections on what they had learned about their ‘self’ through meeting these adolescents. Practitioners’ accounts exemplified a process of expansion of their self-awareness, thus corroborating the importance of reflection on and in action. As a process of self-discovery, self-awareness is embedded in a relationship wherein practitioners constantly revisit their ‘knowing’ and ‘not-knowing’. Referring to her therapeutic work, Sarah clarifies the extent of the commitment to self-awareness:

It certainly challenges [...] all aspects of yourself […], working with them challenges me to go on growing and developing on a personal level as well as a professional level. (Sarah)

Whilst seemingly asserting a composite understanding of the self, Sarah explains that self-awareness demands her whole self. Additionally, the work in complex trauma with adolescents requires Sarah to invest her whole ‘self’ beyond the differentiation between a personal and a professional ‘self’. Through her practice, Sarah would acquire personal maturity.

Consequently, practitioners’ unknown self emerged through an experiential process of becoming one’s self beyond externally imposed expectations, enhancing practitioner’s sense of identity.

### Superordinate Theme 3: Practitioners’ Presence

The last superordinate theme captured participants’ experience of their ‘self’ in their therapeutic work. Participants expressed the importance to be authentic and emanate a sense of safety. This ethical attitude appeared to reinforce practitioners’ commitment to respect their clients’ otherness through empathy and acceptance, whilst embodying compassion as commitment to restoring a sense of justice that had been shattered because of the adolescents’ experiences with complex trauma.

As their clients had been violated through interpersonal relationships, practitioners offered their ‘authentic self’ to demonstrate trustworthiness, embracing the risk of being mutually vulnerable to the other. Accordingly, participants’ experience of their ‘self’ in the therapeutic relationship had the validation of the client’s otherness as a reference point. For instance, “Gordon” pinpoints the qualities of his ‘self’:

There's something about being open and […] surprised […], often you've heard stories before but […] you have to be […] willing to listen to them and consider the individual and not be burnt out when you're doing this kinda job. (Gordon).

Gordon seems to resonate with the idea of abandoning assumptions when encountering the client’s alterity. Consonantly, Gordon can be surprised because he can see the other beyond the trauma and professional expectations. Furthermore, by alluding to compassion fatigue, Gordon warns against the practitioner’s exhaustion which might prevent seeing clients as persons instead of cases. “Have to” suggests that this way of being is a precondition for the therapeutic relationship to develop. Consequently, whilst “Gordon” is ready to welcome his clients, the actualisation of the relational process appears to lie in the other’s power.

Nevertheless, practitioners acknowledged their relational power by expressing their responsibility to set boundaries for the emotional and behavioural dysregulation that adolescents with complex trauma might present. Simultaneously, by tailoring their therapeutic approach in a moment-to-moment process, participants also appreciated their clients’ pace in engaging in the relationship, thus revealing their mutual involvement as persons with needs. Judith’s words appear to capture the essence of the participants’ account:

It's not selfless […], yes it meets my needs too. Hopefully I give more than I get, I love this work […] but it's bloody hard [...]. (Judith)

Judith states that her profession does not imply the abandonment of the practitioner’s self in an act of radical altruism. Working therapeutically means that Judith’s needs are also met through the relationship with the young person. Whilst reclaiming her ‘self’, Judith describes this relationship almost as a transaction whereby the hope is to not objectify the other. As she declares her love for this work, Judith underscores its difficulties almost implying that it requires everything that she can offer, namely her whole ‘self’.

Accordingly, the therapeutic relationship becomes a joint project and shared emotional suffering appears to be inherent to trauma work. However, counterbalancing the seriousness of the work with adolescents and complex trauma, participants also reported to enjoy their practice:

I've got a lot of fun […] to be playful is to be childlike, […] it’s to be able to laugh at yourself […]. It was humour and laughing and being compassionate that got us through all of this and had it not been for that, it would have been too hard work. (Mark)

Mark’s self can still access the primordial experience of playfulness which embodies qualities of innocence, spontaneity, curiosity, and authenticity, perhaps suggesting that the practitioners’ offer of their ‘self’ encompasses their whole developmental history, hinting at the relevance of attachment. Simultaneously, Mark’s sense of humour appears to lighten the emotional intensity of his therapeutic work. Furthermore, by not taking his ‘self’ seriously, Mark seems to relinquish the practitioner’s power to ameliorate the clients’ condition. Thus, Mark would have presumably not coped with an over-focus on suffering, which would overshadow the collaborative nature of a compassionate trauma work that aims to promote resilience.

Consequently, within the power dynamics of the therapeutic relationship, the practitioners’ presence translated into the offer of their authentic and trustworthy ‘self’ to encounter their clients’ otherness.

## Discussion

The results of this study illustrated how the person of the practitioner emerged in trauma work with adolescents through the dynamic experience of the therapeutic relationship, the expansion of practitioners’ self-awareness and their presence as personal involvement beyond their role as professionals.

Firstly, participants reported a distance-relationship dynamic with traumatised young people. As reported by Sleater and Scheiner (2020), mutuality appeared to be a feature of the connection between practitioners and clients, substantiating the role of the real relationship in psychotherapy outcome (Gelso et al. 2018; Wheeler and McElvaney 2018). Over an intrapersonal perspective that would locate the trauma within the young person, participants emphasised how their therapeutic relationship was embedded in a network of relationships, reinforcing the importance of adopting an ecological perspective that includes young people’s wider systemic difficulties (McElvaney and Tatlow-Golden 2016) and practitioners’ organisational context (Edwards and Karnilowicz 2012). Arguably related to their clients’ emotional/behavioural dysregulation and previous experiences of unsafe relationships (Dvir et al. 2014), participants also underscored that being in a relationship means being vulnerable to its rupture, making repairing the therapeutic alliance a component of their work (Eubanks et al. 2018). Specifically, participants voiced a sense of distance from their clients, problematising their connection with young people with experience of complex interpersonal trauma. Whilst therapeutic distance has been explored in adult attachment literature to capture clients’ and therapists’ relational and emotional fluctuations to develop a safe relationship (Daly and Mallinckrodt 2009), this construct remains notably unexplored in child and adolescent research (Mallinckrodt et al. 2015).

Secondly, practitioners’ reported an unknown dimension of their self of which they became aware through their reflection on their practice as a learning experience. Specifically, participants’ account on managing countertransference and seeking supervision adds to the literature that examines the range of emotional, cognitive, bodily, as well as secondary traumatic responses evoked within their therapeutic work (Athanasiadou and Halewood 2011; Hayes et al. 2018; Rosenberger and Hayes 2002). As highlighted by Sleater and Scheiner (2020), participants’ reflection in and on action (Yanow and Tsoukas 2009) was rooted in a relational context, resonating with the concept of “co-transference” (Sapriel 1998, p. 82) that emphasises the role of the practitioners’ person in a co-constructed relationship (Tudor 2018). Whilst countertransference has emerged as a significant construct in trauma work, the parental-protective role reported by participants resonates with vicarious parenting as a specific type of dynamic wherein relational dynamics might intertwine with practitioners’ and adolescents’ attachment styles (Fixsen et al. 2019).

Thirdly, practitioners’ ethical stance shone through their presence with their clients. Whilst exploring the experience of their ‘self’ in the therapeutic relationship, participants shared their involvement beyond their professional role as persons (Baldwin 1987; Gelso et al. 2018; Gendlin 1996; Mearns and Cooper 2017). In line with the recommendation to account for client’s heterogeneity and preferences (Cloitre 2015), participants advocated a tailored approach that respects the client’s pace (Mostowik and Cyranka 2018). As practitioners offered their ‘self’ becoming a healing presence for their clients (Banitt 2018), they also admitted their vulnerabilities, being sensitive to issues of power and social justice that feature trauma work (Afuape 2012). Specifically, practitioners’ touched on playfulness as a way of relating to young people who experienced complex interpersonal trauma, suggesting a way of being that might provide therapeutic benefits (Tait and Wosu 2012) and counteract the effects of vicarious traumatisation (Hidalgo et al. 2016).

### Conclusion

This study engaged with Yardley’s (2000) quality criteria for qualitative research. Demonstrating sensitivity to context (Yardley 2000), this study provides an account of trauma-specialist professionals working with young people. Commitment and rigour (Yardley 2000) were shown through the pilot interview to refine questions and the supervisory process. Transparency and coherence (Yardley 2000) were aided through the supervisor’s contribution and the researcher’s reflexivity. Additionally, this research expands on existing literature combining practitioners’ experiences of trauma work with the use of self (Castonguay and Hill 2017; Malcolm and Golsworthy 2020). Attesting its impact and importance (Yardley 2000), this research contributes to enhancing the understanding of practice as a relational process by elucidating the practitioner’s use of self within trauma work with adolescents. Accordingly, participants’ experiences could inform trauma therapeutic work with young people through a process approach (Schottenbauer et al. 2008).

### Limitations

As the small sample size does not allow generalisation, more studies with a focus on the practitioner’s self in trauma work are required considering diverse populations. Additionally, as practitioner’s use of self is embedded in their relationship with clients, process research might help elucidate dynamics at different points in therapy (Llewelyn and Hardy 2001). Alternatively, a participatory design (Bergold and Thomas 2012) could have counteracted the inherent power imbalance of the researcher-participant relationship (Karnieli-Miller et al. 2009).

### Theoretical Contribution

This study suggests an understanding of the use of self in trauma work. Elaborating on the model of therapeutic use of self by Sleater and Scheiner (2020) within trauma work, an intersubjective conceptualisation of the use of self (Rasmussen 2005) with ethics as foundation (Cooper 2009) may resonate with counselling psychology practice as existential endeavour (Spinelli 2014). The client’s as Other (Larner 2004) faces every practitioner with the yearning to be seen as a unique being (Miller 2008). Firstly, the therapeutic relationship, as ‘human being-with-human being’ is the expression of a twofold movement of distance and relation that realises the person’s relatedness whilst confirming the other’s transcendent alterity (Gendlin 1996). Furthermore, participants’ acknowledgement of their client’s distance attests the emergence of the practitioner’s ‘ethical self’ in the encounter with the other’s transcendence that demands justice (Levinas 1961/1979). Secondly, the practitioner’s self-awareness, as relational endeavour, bonds the vulnerability and the response-ability to the Other’s suffering in a meaning-making process (Frankl 1985; Levinas, 1972/2003). Thirdly, the practitioner’s way of being, whilst offering the Other the opportunity to face alterity, embodies authenticity, whereby being ultimately means caring for the Other (Levinas 1972/2003; Sayre 2005). Thus, clients’ trauma deepens practitioners’ understanding of what being human means because the other’s suffering demands the action of the human community as affirmation of everyone’s right to be (Levinas 1972/2003). Therefore, as acknowledgment of a shared humanness, a respect for otherness and a commitment to reducing suffering, the use of self becomes ‘compassion-in-action’.

### Future Directions

Future trauma-focused research could explore the therapeutic distance and ruptures through a developmental lens whilst exploring the interlinkage of self-awareness, countertransference and attachment in practitioners (Cartwright et al. 2015). Specifically, future studies may be able to detect developmental themes related to the population considered and differences depending on practitioners’ level of training, with a distinction between early clinicians and more seasoned practitioners. Additionally, qualitative studies could provide further insights into the role of the therapist’s playfulness whilst working with traumatised young people and its potential to ameliorate the effects of vicarious traumatisation (Hidalgo et al. 2016). Finally, qualitative research could explore the potential correlation between practitioners’ self-compassion and their ability to resolve therapeutic ruptures. Consequently, a compassionate framework for interventions might urge practitioners to address trauma as a human and societal responsibility (Longden et al. 2016; Vivekananda 2002), thus underlining the socio-cultural implications of this research (Yardley 2000).

To conclude, this study illuminates practitioners’ use of self in trauma work with adolescents, adding to the literature that acknowledges the role of the person of the therapist and suggesting avenues for further research. An intersubjective and ethical model of ‘therapeutic use of self’ is outlined as ‘compassion-in-action’ to call for practitioners’ response-ability to their clients’ trauma as the endeavour of a human and compassionate community.

### Clinical Implications

Practitioners should be aware of how their personal ‘self’ impacts the therapeutic relationship whilst working with traumatised adolescents. Practitioners may explore ‘therapeutic distance’ to manage alliance ruptures/repairs and consider the role of playfulness to counteract the effects of vicarious traumatisation. Further, practitioners should examine how their countertransferential responses may be intertwined with attachment dynamics. Finally, a trauma-specific model is outlined to orientate the conceptualisation of the use of self in psychotherapeutic practice.

## Appendix

### Interview Schedule

1. Can you begin by telling me about your experience of working with complex interpersonal trauma in adolescents?

- How would you describe your modality of working?
- How did you come to choose complex trauma in adolescents as an area to practice?
- What were the key motivations/factors/concerns that you considered?

2) How would you describe yourself in the therapeutic process with this population?

- Having an example in mind: what were the challenges, the positives/negatives? Why?
- What influenced your practice (if anything)?
- How did you relate to clients during the therapeutic relationship?
- How did your way of relating change, if at all?

3) How would you say you used your ‘self’ in your work, if at all?

- Can you give me an example of how you used your ‘self’ in your work with a client?
- What was your experience of bringing yourself into your therapeutic practice?
- What were the challenges/positives, if any?
- How did you promote your therapeutic alliance with your client group?
- What was the impact on the client?

4) What have you learned about yourself?

- How do you feel about working with this population?
- How have you changed, if at all?
- What have you noticed about yourself and your way of relating to clients?
- How did this therapeutic work impact on you, if at all?

5) Is there anything else you want to share?
